# Endophytic Bacterial Communities of *Ginkgo biloba* Leaves During Leaf Developmental Period

**DOI:** 10.3389/fmicb.2021.698703

**Published:** 2021-10-04

**Authors:** Yan Deng, Haonan Huang, Fangying Lei, Shaodong Fu, Kai Zou, Shuangfei Zhang, Xueduan Liu, Luhua Jiang, Hongwei Liu, Bo Miao, Yili Liang

**Affiliations:** ^1^School of Minerals Processing and Bioengineering, Central South University, Changsha, China; ^2^Key Laboratory of Biometallurgy, Ministry of Education, Changsha, China

**Keywords:** flavonoids, bacterial endophyte communities, *Ginkgo biloba*, community assembly, function prediction

## Abstract

Plant-specialized secondary metabolites have ecological functions in mediating interactions between plants and their entophytes. In this study, high-throughput gene sequencing was used to analyze the composition and abundance of bacteria from *Ginkgo* leaves at five different sampling times. The results indicated that the bacterial community structure varied during leaf developmental stage. Bacterial diversity was observed to be the highest at T2 stage and the lowest at T1 stage. Proteobacteria, Firmicutes, Actinobacteria, Chloroflexi, Cyanobacteria, and Bacteroidetes were found as the dominant phyla. The major genera also showed consistency across sampling times, but there was a significant variation in their abundance, such as *Bacillus*, *Lysinibacillus*, and *Staphylococcus.* Significant correlations were observed between endophytic bacteria and flavonoids. Especially, *Staphylococcus* showed a significant positive correlation with quercetin, and changes in the abundance of *Staphylococcus* also showed a strong correlation with flavonoid content. In order to determine the effect of flavonoids on endophytic bacteria of *Ginkgo* leaves, an extracorporeal culture of related strains (a strain of *Staphylococcus* and a strain of *Deinococcus*) was performed, and it was found that the effect of flavonoids on them remained consistent. The predicted result of Tax4Fun2 revealed that flavonoids might lead to a lower abundance of endophytic microorganisms, which further proved the correlation between bacterial communities and flavonoids. This study provided the first insight into the bacterial community composition during the development of *Ginkgo* leaves and the correlation between the endophytic bacteria and flavonoids.

## Introduction

Endophytes, as a group of microorganisms present in the survival history of the host plant, are a variety of endophytic bacteria and fungi that establish a mutualistic beneficial relationship with the host plant (Brader et al., 2017). In order to better adapt to the host plant environment, certain physiological and biochemical characteristics of plant endophytes are assimilated, such as the secondary metabolites (Qin et al., 2009; Cui et al., 2012; Cao et al., 2016). Therefore, endophytes have important application value, such as plant disease resistance and agricultural production (Ramamoorthy et al., 2001; Merzaeva and Shirokikh, 2010; Pham et al., 2017). As an important part of host biology, the research on endophytic bacteria has become a hot topic in recent years. It is therefore necessary to study plant endophytes and their relationship and mechanisms of influence with the host plant.

Many factors can affect the endophytic community of plants, including temperature (Latz et al., 2021), precipitation (Huang, 2020), and soil property (Lehtonen et al., 2005). It has been reported that temporal changes can cause changes in the community composition of the host endophytic bacteria named temporal patterns (Jackson and Denney, 2011). Besides, some studies have shown that the reasons for the change of endophytic microbial community may be related to the change of endophytic environment (Fort et al., 2021). Plants express different functional traits at different stages of growth, including the production of specific secondary metabolites that may guide the assembly of endophytic microbial communities (Chaparro et al., 2014; Wagner et al., 2016). These studies suggest that plant secondary metabolites can mediate interactions between hosts and endophytic microbes. However, the specific influencing factors and mechanisms of endophytic microbial community changes need to be further studied.

*Ginkgo biloba* L. are deciduous trees of *Ginkgo Ginkgoaceae*, which has a high medicinal value. *Ginkgo* extract has been used by the ancients as an herbal remedy for cardiovascular and bronchial diseases (Dubber and Kanfer, 2006; Pereira et al., 2013). Flavonoids as the main active substances in *Ginkgo* are a kind of polyphenolic compound, which have antioxidant and free radical-scavenging effects (Singh et al., 2008). Flavonoids may shape the assembly of endophytic microbial communities. However, most of the studies only focused on the screening of endophytic bacteria and their flavonoid production performance (Yuan et al., 2019), but neglected the exploration of plant endophytes and their relationship with the host plant and flavonoids.

*Ginkgo* leaves contain a large number of active, medicinally valuable compounds. In addition, internal phytochemical profiles vary at various developmental stages in *Ginkgo* leaves. In this study, three wild female *Ginkgo* trees were selected, and the compositional shifts in endophytic bacterial community of wild *Ginkgo* leaves from April to August were assessed. We hypothesized that (i) the composition and diversity of the endophytic bacterial community of *Ginkgo* leaves were variable and patterned with the development of *Ginkgo* leaves and (ii) flavonoids might influence the abundance and community composition of endophytic bacteria in *Ginkgo* leaves. This experiment can support the subsequent search of functional microorganisms and guide the cultivation, protection, and increase of resource utilization of *Ginkgo*.

## Materials and Methods

### Site Description and Sampling Methods

The *Ginkgo* leaf samples were taken from three wild *Ginkgo* trees in 2019 on Yuelu Mountain (28°10′30′′ N, 112°55′20′′ E) in Changsha, Hunan province (Supplementary Table 1). Yuelu Mountain belongs to the subtropical monsoon humid climate area. It is cloudy and rainy in spring and summer. The annual average temperature is 17.2°C, and the annual average precipitation is 1,200–1,400 mm. Yuelu Mountain is a rare “5A” urban mountain scenic area, and the ecological environment of this scenic area is gradually being affected by largely unplanned tourist activity and urbanization. The wild *Ginkgo* trees on the Yuelu Scenic Spot are limited, and the selected three wild *Ginkgo* trees are growing well and protected from damage (namely, S1, S2, and S3). Leaf samples were collected monthly from when the leaves began to grow in April to August. The leaves began to turn yellow in September. The canopy of each tree was divided into three sections, and 200 g of healthy leaves were collected from 5 to 7 m above the ground as one sample from each section. In total, 45 leaf samples were obtained from three trees at five time points (Supplementary Table 2; T1: Apr 1st, T2: May 1st, T3: Jun 1st, T4: Jul 1st, and T5: Aug 1st). To minimize the effects of other factors, all samples were collected aseptically wearing sterile gloves on a sunny day between 9:00 a.m. and 1:00 p.m. Leaves were cut off from each tree, immediately put in a sterile plastic bag, and transported to the laboratory, within 1 h. Environmental temperature and fresh weight of a leaf was recorded at the same time. After being transported to the lab, each sample was divided into three parts for phytochemical analysis, endophyte isolation, and DNA extraction. The parts for DNA extraction were surface sterilized and frozen in liquid nitrogen and then stored at −80°C. The process of surface disinfection was as follows: the sample was firstly flushed cleanly with tap water, then secondly immersed in 75% ethanol for 1 min, thirdly rinsed in sterile deionized water after, fourthly immersed in 8% NaClO for 10 min, then fifthly rinsed in sterile deionized water for five times, and finally dried with sterile filter paper. After surface sterilization, part of the sample was reserved for the isolation of endophytic bacteria, and the rest were triturated in liquid nitrogen and stored at −80°C until DNA extraction. Leaf moisture content was determined by drying the leaves at 70°C to constant weight.

### Phytochemical Analysis of *G. biloba* Leaves

The washed fresh leaves were ground in liquid nitrogen, and 200 mg of the ground powder in liquid nitrogen was added to 30 ml of 70% ethanol, vortexed and homogenized for 1 min, and then subjected to an hour ultrasonic extraction for the flavonoids according to research (Zou et al., 2021). The mixture was centrifuged at 13,000 rpm for 10 min at 4°C and then 1 ml of the supernatant was dried in nitrogen at 37°C. The residue was dissolved in 1 ml of methanol and centrifuged at 13,000 rpm for 10 min at 4°C. The supernatant was passed through a 0.22-μm filter membrane and then diluted to 10 ml. The high-performance liquid chromatography (HPLC) system was used for the determination of the flavonoid content (including quercetin, kaempferol, and isorhamnetin) (Shimadzu, Japan). A C18 column (217 mm × 2.1 mm, 1.7 μm, Waters, Milford, United States) was used for the separation, and the column temperature was controlled at 25°C. Flavonoids were analyzed with 0.05 M sodium acetate (A) and 90% acetonitrile (B) comprising the mobile phase. The gradient elution condition was optimized as follows: 0–20 min, B: 5–70%; 20–23 min, B: 70–5%; 23–25 min, B: 5%. A flow rate of 1.0 ml/min was used. Standard curves were established by a series concentration of the corresponding standard (ChemFaces, Wuhan, China) at 360 nm. Then, the content of total flavonoids was calculated by the following formula (the coefficient in the formula is the average conversion factor between flavonol glycosides and flavonoid glycosides) (Hasler et al., 1992):


T-flavonoids=2.51×quercetin+2.64×kaempferol+2.39×isorhamnetin.

Chlorophyll A and chlorophyll B were determined by acetone decolorization (Arnon, 1949). Malondialdehyde (MDA) in leaves was determined by the thiobarbituric acid method (Reitznerova et al., 2017). Superoxide dismutase (SOD) activity from leaf samples was extracted using Plant tissue Cu^2+^Zn^2+^—SOD assay kit (Nanjing, Jiancheng, China).

### DNA Extraction, Sequencing, and Analysis of the Endophytic Bacterial Community in *G. biloba* Leaves

Genomic DNA of endophytic bacteria from leaf samples was extracted using DNeasy^®^ Plant Mini Kit (250) (Qiagen, Inc., Dusseldorf, Germany). The V4–V5 hypervariable regions of the 16S rDNA were amplified using the 799F (5′-AACMGGATTAGATACCCKG-3′)/1115R (5′-AGGGTTGCGCTCG TTG-3′) (Chelius and Triplett, 2001; Redford and Fierer, 2009). The amplified DNA was determined by running the samples on a 1.2% agarose gel and then purifying using the Gel Extraction Kit D2500 (OMEGA Bio-tek, Norcross, Georgia, United States).

High-throughput sequencing of the library preparation products was conducted on an Illumina Hiseq platform (Hiseq PE250). Gene sequencing data of raw 16S rRNA were processed using the Galaxy pipeline established by Zhou’s lab^1^ at Oklahoma University (OU), United States. To ensure the quality of the sequences, both forward and reverse reads were trimmed based on sequence quality scores using Btrim (Kong, 2011; Deng et al., 2012). After trimming, the forward and reverse reads with 50–250 bp overlapping were combined to obtain longer sequences. Also, unqualified sequences were removed if they were too short or contained an undetermined base “N.” Following this, potential chimeric sequences were detected and removed by UCHIME. Sequences were then clustered into operational taxonomic units (OTUs) at 97% sequence similarity using UCLUST (Edgar et al., 2011). Finally, the RDP Classifier was used to assign 16S rRNA sequences to the bacterial taxa^2^ with a minimal 50% confidence estimate. Also, it was necessary to filter OTUs classified as mitochondrion or chloroplast in data outputted from Galaxy pipeline and those unclassified OTUs (Li and Godzik, 2006; Wang et al., 2007). All sequences produced from Illumina sequencing had been uploaded to the sequence read archive (SRA) of NCBI database with numbers from SRR13946092 to SRR13946136.

Alpha diversity indexes, including Shannon index (*H*) and Pielou evenness (*J*), were calculated on Institute for Galaxy| Denglab pipeline.^3^ Non-metric multidimensional scaling (NMDS) was used for observing the changes of bacterial community composition along with the leaf growth. The analysis of similarities (ANOSIM) was performed to analyze the significance of clustering. Redundancy analysis (RDA), variance partitioning analysis (VPA), and Mantel test were carried out on the R platform, with the “Vegan” Package, for correlating between bacterial community and all the environmental factors. For RDA and VPA, correlated variables were removed [variance inflation factor (VIF) > 10] using variance inflation factors test. Tax4Fun2 was used to predict the relative abundance of functional genes of each sample (Asshauer et al., 2015).

### Isolation of Endophytes in *G. biloba* Leaves and Their Flavonoid Resistance

The surface-sterilized leaves were physically crushed using phosphate-buffered saline (PBS). Then, the suspension obtained was spread on R2A medium agar plates (0.5 g/l yeast extract, 0.5 g/l peptone, 0.5 g/l casein hydrolysate, 0.5 g/l glucose, 0.5 g/l soluble starch, 0.3 g/l dipotassium hydrogen phosphate, 0.024 g/l anhydrous magnesium sulfate, 0.3 g/l sodium pyruvate, and 15 g/l agar). All colonies were streaked for consecutive times, and finally, 11 bacterial strains were obtained. The identification of the strains was carried out by BIG (Shenzhen, China). The sequences obtained were analyzed using NCBI BLASTn search. Among them, two strains, including a strain of *Staphylococcus* and a strain of *Deinococcus*, were selected for flavonoid resistance analysis based on a correlation analysis between endophytic bacteria and flavonoids. The isolates were inoculated in 50 ml R2A medium at 30°C and 200 rpm to obtain the seed liquid with OD_600_ ≈ 0.9. The seed liquid was then diluted 100-fold with fresh R2A medium containing a gradually increased concentration of flavonoids according to the concentration of flavonoids in leaves. Total flavonoid content (quercetin/kaempferol/isorhamnetin = 1:2:1) was uniformly set: 0, 152.7, 305.4, 458.1, and 610.8 mg/l. The absorbance of bacterial liquid (OD_600_) was measured every 6 h.

## Results

### Phytochemical Characteristics of *G. biloba* Leaves in Leaf Developmental Period

During the annual developmental period, the average weight of *Ginkgo* leaves increased gradually from April to August (Supplementary Table 3). The fresh weight of a leaf was 0.37–0.46 g at the T1 stage and increased rapidly by 0.18–0.21 g at the T2 stage and 0.17–0.26 g at the T3 stage. Then, the fresh weight of *Ginkgo* leaves remains relatively stable at T4 and T5. Thus, based on changes in leaf weight, we divided the growth process of the leaves into three stages, namely, T1 stage: leaf bud formation; T2 and T3 stages: rapid growth stage of leaves; and T4 and T5 stages: late growth stage of leaves.

**FIGURE 1:**
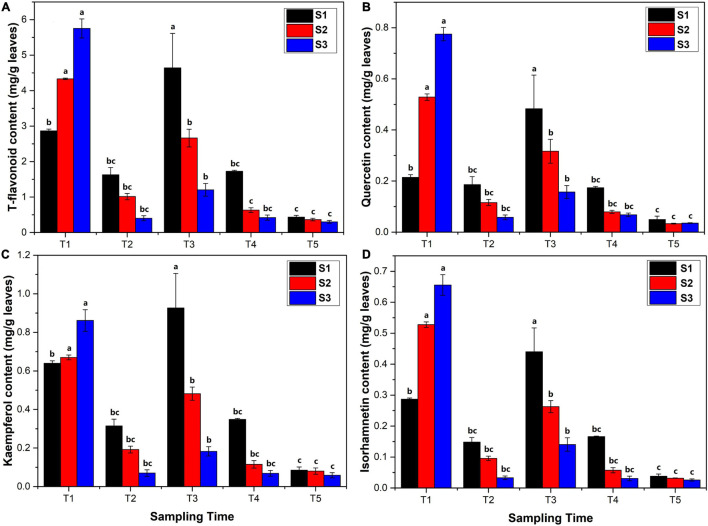
Flavonoid content (mg/kg leaves) analysis in *Ginkgo biloba L.* extracts determined by high-performance liquid chromatography (HPLC) using gradient elution at 360 nm. **(A)** The content of total flavonoids. **(B)** The content of quercetin. **(C)** The content of kaempferol. **(D)** The content of isorhamnetin. The error bar was plotted based on the standard deviation of replicate samples.

The content of T-flavonoids, quercetin, kaempferol, and isorhamnetin, in *Ginkgo* leaves were determined by HPLC analysis (Figure 1). The results showed that the T-flavonoid content was 2.864–5.753 mg/g in the new leaves at the T1 stage (Figure 1A), followed by a sharply decreased trend at the T2 stage (0.404–1.630 mg/g) with the rapid growth of leaves. The content of flavonoids significantly increased to 1.078–5.764 mg/g in June at the T3 stage and then decreased gradually to 0.434–0.301 mg/g at the T5 stage. The temporal variation pattern of quercetin, kaempferol, and isorhamnetin content was similar to T-flavonoid content. The quercetin content was 0.202–0.791 mg/g in April, 0.049–0.222 mg/g in May, 0.139–0.635 mg/g in June, 0.062–0.179 mg/g in July, and 0.031–0.065 mg/g in August, respectively (Figure 1B). The kaempferol content was 0.626–0.926 mg/g in April, 0.049–0.355 mg/g in May, 0.165–1.132 mg/g in June, 0.056–0.352 mg/g in July, and 0.049–0.103 mg/g in August, respectively (Figure 1C). The isorhamnetin content was 0.282–0.693 mg/g in April, 0.026–0.165 mg/g in May, 0.125–0.529 mg/g in June, 0.026–0.167 mg/g in July, and 0.023–0.046 mg/g in August, respectively (Figure 1D).

**FIGURE 2:**
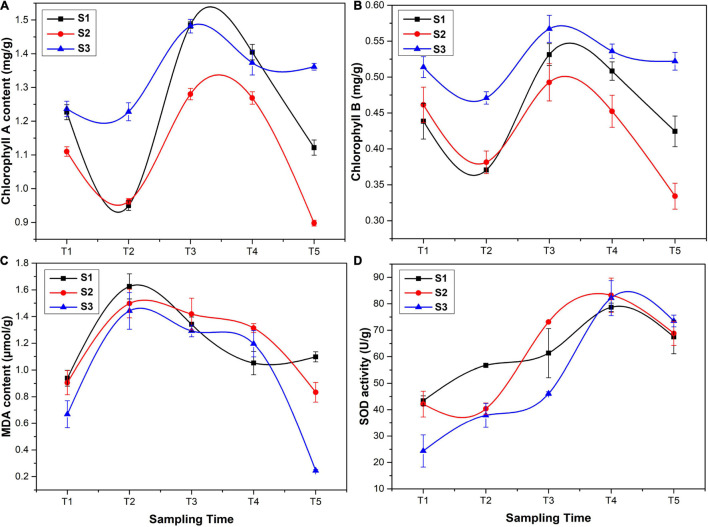
Phytochemical analysis of *G. biloba* leaves: **(A)** chlorophyll A content, **(B)** chlorophyll B content, **(C)** malondialdehyde (MDA) content, and **(D)** superoxide dismutase (SOD) activity.

The results of phytochemical analysis of *Ginkgo* leaves are shown in Figure 2. The chlorophyll A and chlorophyll B content was firstly decreased in May, then reached the maximum in June, and gradually declined in July and August, which was similar to the changes of flavonoid content (Figures 2A,B). The maximum content of chlorophyll A and chlorophyll B at the T3 stage was 1.280–1.486 and 0.493–0.531 mg/g, respectively. MDA is a crucial product of membrane lipid peroxidation, and its accumulation can aggravate damage and lead to aging and resistance physiology (Liu et al., 2021). The MDA content was first increased at the T2 stage and then declined. The highest content of MDA was 1.442–1.626 μmol/g at the T2 stage (Figure 2C). SOD is considered as one of the most effective antioxidant enzymes for all the aerobic organisms, which catalyzes and reduces O^2–^ to H_2_O_2_, thus minimizing the risk of hydroxide (OH^–^) formation (Liling et al., 2021). The activity of SOD showed an increasing trend with the maximum value at the T4 stage (78.671–83.245 U/g) (Figure 2D), followed by a slight decrease at the T5 stage.

### Endophytic Bacterial Diversity and Community Composition in *G. biloba* Leaves

**FIGURE 3:**
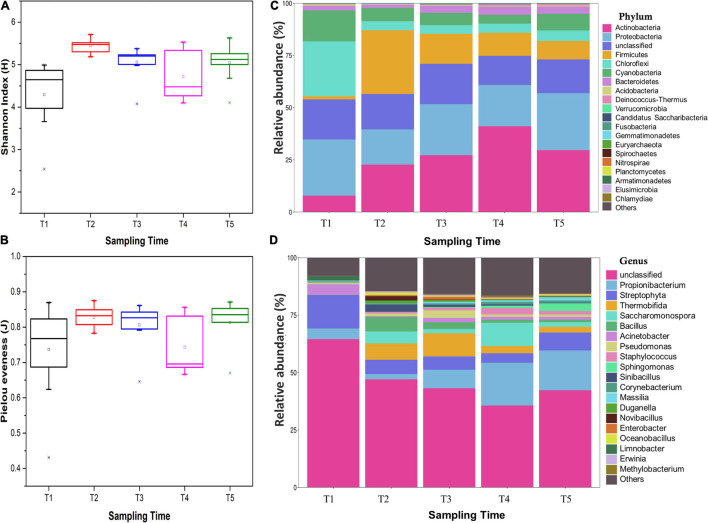
Diversity indexes of endophytic bacterial communities within an individual tree: **(A)** Shannon index, **(B)** Pielou evenness, **(C)** endophytic bacterial relative abundance at the phylum level, and **(D)** endophytic bacterial relative abundance at the genus level. All indexes were means with standard deviations (SDs) calculated from nine replicates. The different lower case letters following the indexes indicate a significant difference (*P*< 0.05) between trees.

**FIGURE 4:**
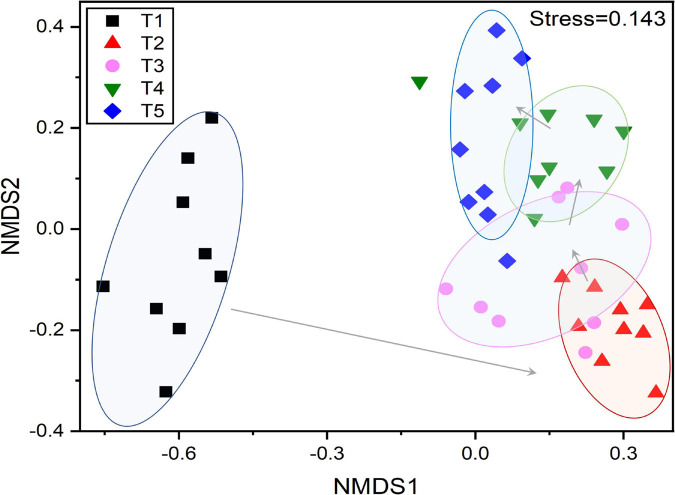
Non-metric multidimensional scaling (NMDS) based on Bray–Curtis distances showing the changes in the composition and β-diversity of the endophytic bacterial community in *G. biloba* leaves at different development stages.

A total of 116,218 high-quality 16S rRNA gene sequences were obtained. By UCLUST at the 97% similarity, 576 OTUs were obtained based on 16S Greengene database, and the rarefaction curves showed that the sequencing depth was sufficient for further analysis (Supplementary Figure 1). Venn diagram showed that all the samples shared 20 core OTUs (Supplementary Figure 2). The results of alpha diversity indexes showed that the Shannon index and Pielou evenness of endophytic community in leaves were slightly lower at the T1 stage and then kept a relatively stable level at T2–T5 stages (Figures 3A,B). NMDS analysis indicated that the bacterial communities in leaves were dynamic and time-varying (Figure 4). Temporal patterns were obvious. The replicates of leaf samples at the same time point were well clustered. The ANOSIM test on Bray–Curtis matrix also showed that the endophytic communities were significantly different among the T1–T5 groups (Supplementary Table 4, *p* < 0.05). Most notably, the endophytic communities at the T1 stage were completely separated from the other four groups on the first axis, and the endophytic communities at the other four stages were gradually changed along with the second axis.

The endophytic bacterial community in *Ginkgo* leaves was mainly composed of Proteobacteria (10.42–43.98%), Firmicutes (0.86–38.42%), Actinobacteria (4.19–49.22%), Chloroflexi (1.80–48.97%), Cyanobacteria (3.04–21.18%), and Bacteroidetes (0.69–5.70%) (Supplementary Figure 3 and Supplementary Table 5). The average relative abundance of endophytic bacterial community at phylum level is shown in Figure 3C. Most of those phyla showed different abundance in different groups, e.g., the phyla Actinobacteria was significantly increased from the T1 stage (7.86%) to the T4 stage (41.09%) and then decreased at the T5 stage (29.65%). Chloroflexi and Cyanobacteria were significantly more abundant in the T1 group (26.2 and 14.84%, respectively) than the other four groups (4.12–5.00% and 4.25–7.99%, respectively), while Firmicutes showed a significantly lower relative abundance in T1 samples (1.60%). Then, Firmicutes was gradually decreased from the T2 stage (30.49%) to the T5 stage (8.84%). When referring to genus level, 576 genera were identified from all sequences and the most abundant 20 genera were shown (Figure 3D and Supplementary Figure 4). The main genera were *Acinetobacter* (0.28–12.77%), *Bacillus* (0.02–7.99%), *Propionibacterium* (1.44–32.29%), *Pseudomonas* (0.07–6.26%), and *Staphylococcus* (0.11–6.97%). Most of those genera in T1 samples had significantly different abundances from others. For example, the genera *Bacillus*, *Saccharomonospora*, *Sinibacillus*, and *Thermobifida* were less than 0.1% in the bacterial community of T1 samples, while they reached over 3.17–10.08% in T2 and T3 samples and then decreased to 0.24–2.53% in T5 samples. Genera *Streptophyta* and *Acinetobacter* were significantly higher in the T1 group (14.62 and 4.45%, respectively) than the other four groups (4.13–7.82% and 0.37–1.90%, respectively). In addition, some genera, including *Propionibacterium*, *Sphingomonas*, *Corynebacterium*, *Massilia*, and *Staphylococcus*, were gradually increased from the T1 stage to T5 stage. These results indicated that changes in endophytic bacterial community composition accompanied the development of the leaves.

### Linkages Between Phytochemical Parameters and Endophytic Bacterial Abundances in *G. biloba* Leaves

The relationship between endophytic bacterial populations and flavonoids as well as physicochemical parameters was analyzed by the Spearman correlation test and RDA. The phylum Actinobacteria was significantly negatively correlated with three flavonol glycosides, while Cyanobacteria and Chloroflexi were significantly positively correlated with three flavonol glycosides (Table 1, *P* < 0.05). Actinobacteria was positively correlated with chlorophyll B, SOD, and temperature and negatively correlated with leaf water content (Table 2, *P* < 0.05). Cyanobacteria showed an opposite relationship with chlorophyll B, SOD, temperature, and leaf water content. Firmicutes was positively related to MDA (*r* = 0.74, *p* < 0.001), and Bacteroidetes was positively related to SOD and temperature. At genus level, most of the genera, including *Aerococcus*, *Blastococcus*, *Chryseobacterium*, *Deinococcus*, *Lysinibacillus*, *Methylobacterium*, *Saccharomonospora*, etc., were significantly positively correlated with three flavonol glycosides, while *Staphylococcus* and *Propionibacterium* were negatively correlated with three flavonol glycosides (Supplementary Table 6, *P* < 0.05). In addition, most of the genera were mainly negatively influenced by SOD and MDA and positively influenced by temperature (Supplementary Table 7).

**TABLE 1:** Spearman correlation test showing the relative abundance of endophytic bacterial phyla association with three flavonol glycosides.

Phylum	Quercetin		Kaempferol		Isorhamnetin	
	*r*	*p*	*r*	*p*	*r*	*p*
Acidobacteria	0.093	0.544	0.111	0.468	0.170	0.264
Actinobacteria	**–0.537**	**0.000**	**–0.574**	**0.000**	**–0.600**	**0.000**
Bacteroidetes	–0.050	0.746	–0.110	0.471	–0.147	0.333
Candidatus saccharibacteria	–0.030	0.849	–0.058	0.705	–0.002	0.989
Chloroflexi	**0.304**	**0.043**	**0.372**	**0.012**	**0.432**	**0.003**
Cyanobacteria	0.286	0.057	**0.319**	**0.033**	**0.333**	**0.026**
Deinococcus–thermus	–0.009	0.955	–0.014	0.925	0.052	0.734
Firmicutes	–0.147	0.334	–0.115	0.452	–0.220	0.142
Fusobacteria	–0.034	0.823	–0.075	0.622	–0.025	0.872
Gemmatimonadetes	–0.014	0.930	–0.043	0.780	–0.023	0.882
Nitrospirae	–0.043	0.780	–0.079	0.605	–0.025	0.872
Proteobacteria	–0.115	0.451	–0.126	0.408	–0.079	0.603
Verrucomicrobia	–0.017	0.913	–0.049	0.750	0.009	0.954

*Significant differences (P < 0.05) are indicated in bold.*

**TABLE 2:** Spearman correlation test showing the relative abundance of endophytic bacterial phyla association with physical and chemical parameters.

Phylum	MDA	Chlorophyll A	Chlorophyll B	SOD	Water	Temperature
Acidobacteria	–0.265	–0.034	–0.113	–0.052	0.018	–0.121
Actinobacteria	0.205	0.284	0.353*	0.657***	−0.507***	0.631***
Bacteroidetes	–0.272	0.128	0.120	0.525***	–0.213	0.376*
Candidatus saccharibacteria	–0.056	0.030	–0.003	–0.054	0.146	0.052
Chloroflexi	−0.242**	–0.000	–0.059	–0.143	0.457**	−0.581***
Cyanobacteria	–0.387	–0.266	−0.313*	−0.356*	0.369*	−0.543***
Deinococcus–Thermus	–0.238	0.286	0.259	0.342*	–0.122	0.233
Firmicutes	0.740***	0.013	0.083	0.064	–0.125	0.122
Fusobacteria	–0.130	0.076	0.050	0.054	–0.086	0.186
Gemmatimonadetes	0.011	0.009	–0.012	–0.052	–0.106	0.103
Nitrospirae	–0.084	–0.012	–0.038	–0.085	–0.29	0.106
Proteobacteria	–0.468	–0.046	–0.097	–0.048	0.008	0.019
Verrucomicrobia	–0.113	–0.004	–0.034	–0.072	0.014	–0.236

*Significant differences are indicated in bold.*

*Indicates significant differences (P < 0.05);

**indicates significant differences (P < 0.01);

***indicated significant differences (P < 0.001).

**FIGURE 5:**
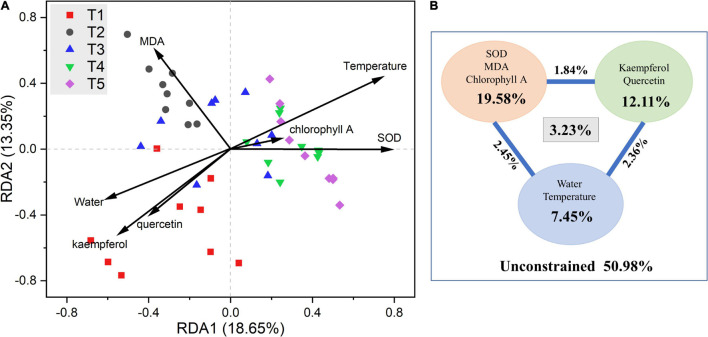
**(A)** Redundancy analysis (RDA) and **(B)** variance partitioning analysis (VPA) of the relationships between endophytic bacterial community and environmental variables in *G. biloba* leaves.

RDA and VPA plots after removing all correlated variables are shown in Figure 5. Among which, chlorophyll B, T-flavonoid, and isorhamnetin were removed because of their VIF > 10. The results of the RDA showed that six of the seven selected physicochemical variables were significantly correlated with the microbial community structure (Supplementary Tables 8, 9). Kaempferol, quercetin, and leaf water content mainly influenced the microbial community structure in the T1 period. Endophytic bacterial communities in T2 and T3 periods were mainly impacted by MDA, and bacterial communities in T4 and T5 periods were mainly impacted by temperature and SOD. For VPA, variables were classified into three groups: flavonoids (kaempferol and quercetin), phytochemical properties (SOD, MDA, and chlorophyll A), and environmental parameters (leaf water content and temperature). VPA showed that these seven variables constrained 49.12% of the total community distribution. The flavonoids constrained 12.11% of the community distribution, while phytochemical properties and environmental parameters constrained 19.58 and 7.45%, respectively.

### Isolation of Endophytes in *G. biloba* Leaves and Their Flavonoid Resistance

**FIGURE 6:**
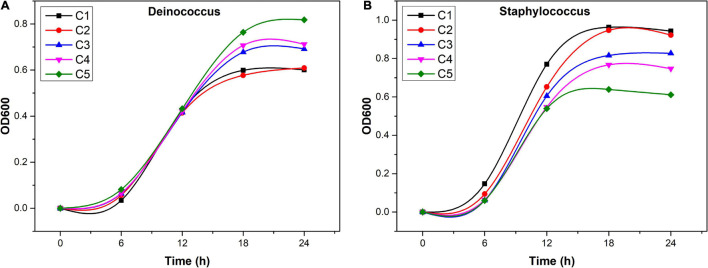
Growth of *Staphylococcus* **(A)** and *Deinococcus* **(B)** in R2A medium with additional flavonoids (C1: 0 mg/l, C2: 152.7 mg/l, C3: 305.4 mg/l, C4: 458.1 mg/l, and C5: 610.8 mg/l).

In total, 11 isolates were obtained, including *Microbacterium*, *Deinococcus*, *Paenibacillus*, *Bacillus*, *Staphylococcus*, *Achromobacter*, *Burkholderia*, and *Massilia*, and the specific details are shown in Supplementary Table 10. Among which, only *Deinococcus* and *Staphylococcus* were shown to significantly correlate with at least one of the three flavonol glycosides (Supplementary Table 6). *Deinococcus* was positively correlated with three flavonol glycosides, and *Staphylococcus* was negatively correlated with quercetin and other two flavonol glycosides (not significant). We monitored the growth of selected endophytic strains in R2A media supplemented with flavonoids (Figure 6). After 18-h culture, *Deinococcus* and *Staphylococcus* reached stationary growth phase. The maximum OD_600_ of *Deinococcus* increased from 0.58 (0 mg/l) to 0.85 (610.8 mg/l) with the increase of flavonoids, while OD_600_ of *Staphylococcus* decreased from 0.97 (0 mg/l) to 0.59 (610.8 mg/l) with the increase of flavonoids.

### Functional Gene Prediction by Tax4Fun2

**FIGURE 7:**
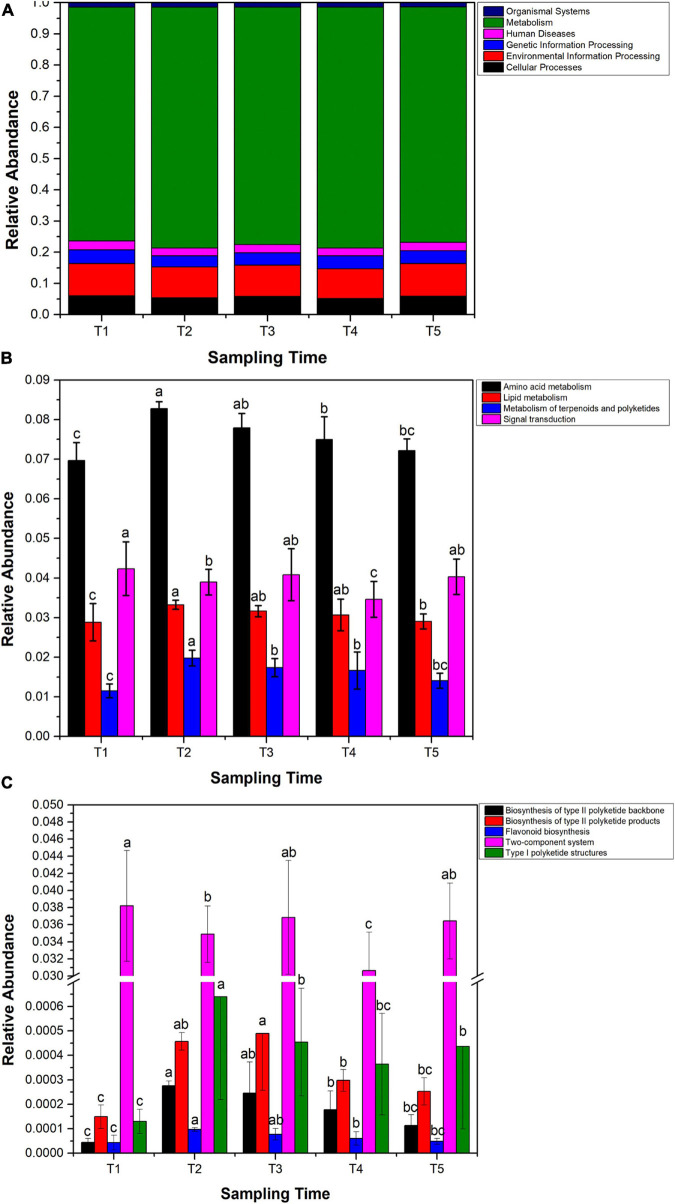
Functional genes analyzed by Tax4Fun2. **(A)** Relative abundance of predicted functional genes at level I. **(B)** Relative abundance variation of genes involved in amino acid metabolism, lipid metabolism, metabolism of terpenoids and polyketides, and signal transduction. **(C)** Changes of relative abundance of predicted genes in the polyketone metabolism, flavonoid biosynthesis, and two-component systems. Samples with different letters indicate that there are significant differences between samples (*p* < 0.05).

By gene function analysis, seven ortholog taxa at level I had been annotated in the *Ginkgo* endophytic community. The relative abundance of these ortholog taxa tended to be similar in five sample groups. Most of these function genes were metabolism, accounting for nearly 75% of all genes (Figure 7A). At level II, the relative abundance of four functional genes including amino acid metabolism, lipid metabolism, metabolism of terpenoids and polyketides, and signal transduction reached the highest at the T2 stage, and then gradually decreased until T5. However, the relative abundance of signal transduction showed an almost opposite trend (Figure 7B). Further analysis at level III showed related genes of polyketide, including biosynthesis of type II polyketide backbone, biosynthesis of type II polyketide products, flavonoid biosynthesis, and type I polyketide structures, reaching the highest abundance at the T2 stage and then gradually decreasing from the T2 stage to T5 stage.

## Discussion

### Leaf Developmental Stage Strongly Influenced Phytochemical Characteristics in *G. biloba* Leaves

*Ginkgo* is one of the most important medicinal plants, and flavonoids are the main medicinal active substances in *Ginkgo*. According to our results, the content of three flavonol glycosides rapidly changed along with leaf developmental stages. Previous studies showed that the synthesis and accumulation of secondary metabolites in *Ginkgo* were a temporal variational process (Lin et al., 2020; Wang et al., 2020; Tetali et al., 2021). This temporal variational process may be related to the growth differentiation balance (GDB) of plants (Herms, 1999; Karasov et al., 2017). The temporal pattern of flavonoid variation in *Ginkgo* leaves was the result of competition between resources for growth and resources for defense. Based on changes in flavonoid content and the increase in biomass of *Ginkgo* leaves, it was found that there was a sudden decline in flavonoid content during the phase of rapid growth of *Ginkgo* leaves at T1–T2 stages and a rise in flavonoid content when growth slowed down at T2–T3 stages. Later when *Ginkgo* is starting to bear fruit, most of the resources obtained was used for the growth of the fruit, which led to a steady decrease in flavonoid content (Lindroth and Peterson, 1988; Lin et al., 2020).

As a reference value for the temporal variation in flavonoid content of *Ginkgo* leaves, the variation in physicochemical parameters aided in explaining the reason for the variation in flavonoids. The increased chlorophyll content enhanced the photosynthesis intensity, resulting in an increase in flavonoid content to some extent (Torres et al., 2021). The antioxidant mechanism of *Ginkgo* explains to some extent the reasons for the relevant changes in in flavonoid content (in terms of changes in MDA content and SOD activity). The antioxidant response of *Ginkgo* leaves was mainly non-enzymatic antioxidant first and followed by enzymatic reactions in the early stage, which resulted in the higher flavonoid content and lower SOD activity (Li et al., 2021). With the growth of *Ginkgo* leaves, rapid changes in flavonoid content were due to the depletion of flavonoids and the large allocation of resources to leaf growth. Coupled with changes in ambient temperature and leaf water content, plant cell membrane oxidation increased, leading to an increase in MDA content, while SOD activity increased with the antioxidant response mechanism (Baisak and Acharya, 1994).

### Leaf Developmental Stage Strongly Influenced the Assembly of Endophytic Microbiome in *G. biloba* Leaves

The study of endophytic bacteria in *Ginkgo* leaves is of great value to the follow-up study of medicinal components and the exploitation of medicinal component-producing microbial resources. Our results demonstrate that endophytic microbiome assembly in *Ginkgo* leaves was mainly influenced by leaf developmental stage regardless of individual species (Figure 4). The regionality caused the formation of core members of endophytic bacteria in the host plant, but developmental stages caused substantial changes in endophytic bacterial diversity (Figure 3) and community structure (Figure 4) in *Ginkgo* leaves (Huang, 2019). Because for individual species, the effect of time on endophytic bacterial community assembly was much stronger than spatial locations (Figure 4), although spatial locations are well-known to make a great contribution to bacterial community composition (Meyer and Leveau, 2012; Leff et al., 2015). It was suggested that time itself usually is not a driver of community assembly processes (Hui et al., 2017). Microbial communities, like plant and animal communities, are dynamic and exhibit temporal patterns that can reflect underlying biotic and abiotic processes (Banning et al., 2011; Blasko et al., 2015; Wu et al., 2015). On the other hand, the functional capacity of bacterial species is key to their recruitment by hosts, and Burke et al. (2011) proposed that bacterial community assembly is associated with function rather than the taxonomy (Burke et al., 2011; Grady et al., 2019). For example, changes in the abundance of *Bacillus* demonstrated its growth-promoting effect, as the proportion of *Bacillus* increased rapidly during the rapid growth period of *Ginkgo* leaves (Compant et al., 2005). *Lysinibacillus* is a genus of endophytic bacteria capable of inducing defense responses in host plants. However, the results showed that it was not found in the samples of T1. This could be that *Lysinibacillus* belongs to a group of soil bacteria that are usually found in roots and therefore would not have been found in the early leaves (Passera et al., 2021). Furthermore, based on the results of alpha diversity analysis and beta diversity analysis, the endophytic bacterial community of *Ginkgo* leaves was not a temporally linear succession process. Therefore, it suggested that certain factors dominated the assembly of the endophytic bacterial community under temporal turnover, such as secondary metabolites. Moreover, this influential nature decreased with time, and changes in the endophytic bacterial community stabilized, which is consistent with the results we obtained (Grady et al., 2019).

### Flavonoids Attributed to the Assembly of Endophytic Bacterial Communities in *G. biloba* Leaves

Secondary metabolites are unique natural compounds that can, to some extent, guide the assembly of specific bacterial communities (Sasse et al., 2018; Stringlis et al., 2018). A shred of evidence showed that salicylic acid causes alterations in the response of bacterial communities in the *Arabidopsis* root zone to signals (Lebeis et al., 2015). A comparison of bacterial profiles in *Arabidopsis* roots also shows that the triterpene biosynthetic network can regulate the establishment of specific microbiota (Lebeis et al., 2015; Huang et al., 2019). In this study, both phytochemical characteristics and endophytic bacterial community of the *Ginkgo* leaves varied with developmental stages. It was suggested that phytochemical characteristics of the host *Ginkgo* affected the endophytic bacterial community. For example, the genus *Staphylococcus*, as in the results of this study, was low in abundance in the pre-growth phase and increased in abundance in the late growth phase of *Ginkgo* leaves, because of the effect of changes in flavonoid content (Osorio et al., 2021). Otherwise, the results of the alpha diversity analysis of the endophytic bacterial community of *Ginkgo* leaves showed that the factors influencing the diversity of the endophytic bacterial community were related to flavonoids, as the trends of changes in the two clearly corresponded to the opposite. Moreover, the results of the beta diversity analysis showed that the endophytic communities in the early stages were more different, which was caused by the greater variation in flavonoid content in the early stages. The results for RDA were similar, with the endophytic bacterial community being most associated with flavonoids in the early stages, and its influence diminishing over time (Figure 5). This weakening effect over time confirmed the result that flavonoids could influence the assembly of endophytic bacterial communities in *Ginkgo* leaves.

Host plant metabolites can also selectively regulate the growth of bacteria from different taxa by acting as antibiotics or proliferating agents, and plant exudation traits and microbial substrate uptake traits could interact to yield specific patterns of microbial community assembly (Zhalnina et al., 2018). For example, scopoletin selectively inhibits soil-borne fungal pathogens, while growth-promoting rhizobacteria is highly tolerant to scopoletin (Stringlis et al., 2018). Flavonoids selectively inhibit the growth of bacteria by acting as antibiotics (Cushnie and Lamb, 2005). As shown by the results of the Spearman correlation analysis, flavonoids did have an inhibitory effect on the genera of bacteria that were significantly negatively correlated with flavonoids in this study, such as *Propionibacterium*, *Saccharomyces*, and *Staphylococcus* (Supplementary Table 6). However, some microbes are highly tolerant to antibiotics and join forces to trigger flavonoid metabolism (Cui et al., 2012; Cao et al., 2016), as we observed that some genera, including *Deinococcus*, *Propionibacterium*, *Saccharomonospora*, *Staphylococcus*, etc., had significant correlations with flavonol glycosides in this study (Supplementary Table 6). Our culture results showed that the increase of flavonoid content could promote the accumulation of *Deinococcus* (Figure 6A), which might be the result of amylosucrase produced by *Deinococcus* being able to utilize quercetin (Rha et al., 2020). Moreover, *Deinococcus* was also found to produce flavonoid, which may contribute to the anti-oxidative damage ability of this species (Sajjad et al., 2017). Instead, the inhibitory effect of flavonoids on *Staphylococcus* was found to be enhanced with the increase of concentration (Figure 6B). Previous studies speculated that the inhibitory effect of flavonoids might be realized by interfering with the entry of nutrients and metabolites into bacteria (Tanaka et al., 2004). The results suggested that flavonoids play an important role in the assembly of endophytic communities (Figure 5).

Generally speaking, microorganisms select for their environment mainly in terms of the set of functions (Hammesfahr et al., 2011). Therefore, functional predictions were made about endophytic bacterial communities and found that flavonoids affected endophytic bacterial community assembly at the molecular level (Figure 7). Based on the results of temporal changes in flavonoid content and temporal changes in the abundance of certain genes in the endophytic bacterial community (Figures 7B,C), it can be hypothesized that the microbes colonized in the plant might be affected by flavonoids produced by the host. In general, environmental stress is an important reason for the high abundance of early microbial environmental information-processing genes (Debroas et al., 2009). Signal transduction, an important molecular response mechanism for cells to process stimulus signals, is one of the means by which bacteria process environmental information (Cascales et al., 2014). In this study, the results revealed that the abundance of signal transduction genes in the endophytic bacterial community of *Ginkgo* leaves was higher in the pre-growth phase of *Ginkgo* leaves. This may be the result of a response to environmental stress in the pre-growth phase of *Ginkgo* leaves, including the effect of high concentrations of flavonoids. Amino acid metabolism and lipid metabolism are metabolic processes that are essential for the survival of microorganisms (Cai et al., 2012; Parsons and Rock, 2013). The results of this study found that the trend in gene abundance for these two metabolisms in the endophytic bacterial community of *Ginkgo* leaves was opposite to the trend in flavonoid content. This is consistent with the report of Zeng et al. (2020) that flavonoids can regulate endophytic bacterial communities by inhibiting amino acid metabolism and lipid metabolism in endophytic bacteria. Endophytes coexist with their hosts, and they adapt to each other and coevolve. It has been shown that genes and abilities that evolve in one lineage are usually acquired steadily by another lineage (Papke and Gogarten, 2012). Direct gene transfer between species has occurred in all major taxa and seems to occur more frequently in prokaryotes (Moran, 2007). Genes including flavonoid synthesis and polyketide metabolism were observed in endophytic bacteria of *Ginkgo* leaves. The results showed that flavonoid synthesis in the host likely influences the expression of genes related to flavonoid synthesis in the endophytic bacteria. It is like a complementary process (Beekwilder et al., 2007; Ma et al., 2009).

## Conclusion

This study provided the first insight into the bacterial communities at different growth stages in *Ginkgo* leaves. There were significant differences in bacterial communities among different times under the influence of different growth stages of *Ginkgo* leaves and flavonoids. A strong correlation between the endophytic bacteria and flavonoids was found by the Spearman correlation analysis. *In vitro* cultivation experiments further provided evidence for the effects of flavonoids on microorganisms. The predicted results of Tax4Fun2 further demonstrated that flavonoids might affect the variation of bacterial communities in *Ginkgo* leaves at different growth periods. All in all, this experiment can support the subsequent search of functional microorganisms and guide the cultivation, protection, and increase of resource utilization of *Ginkgo*.

## Data Availability Statement

The datasets presented in this study can be found in online repositories. The names of the repository/repositories and accession number(s) can be found below: https://www.ncbi.nlm.nih.gov/, SRR13946092–SRR13946136.

## Author Contributions

YD, HH, KZ, SF, SZ, YL, and XL conceived and designed the work. YD, HH, KZ, SF, and FL performed the experiments. YD and HH analyzed the data and wrote the manuscript. YD revised the manuscript, reanalyzed the data, and plotted. All authors contributed to the article and approved the submitted version.

## Conflict of Interest

The authors declare that the research was conducted in the absence of any commercial or financial relationships that could be construed as a potential conflict of interest.

## Publisher’s Note

All claims expressed in this article are solely those of the authors and do not necessarily represent those of their affiliated organizations, or those of the publisher, the editors and the reviewers. Any product that may be evaluated in this article, or claim that may be made by its manufacturer, is not guaranteed or endorsed by the publisher.

## Footnotes

1
http://zhoulab5.rccc.ou.edu/

2
http://rdp.cme.msu.edu/classifier/classifier.jsp

3
http://mem.rcees.ac.cn:8080/
