# Supplementary material for: Endophytic Bacterial Communities of Ginkgo biloba Leaves During Leaf Developmental Period
Source: Front Microbiol. 2021 Oct 4;12:698703. doi: 10.3389/fmicb.2021.698703 (PMC8521191; doi:10.3389/fmicb.2021.698703)

Supplementary Material

# Supplementary Tables

**Table S1.** The locations of the sample trees.

| Plot | Label | Longitude | Latitude | Altitude | Height | Diameter | Sunnyside |
| --- | --- | --- | --- | --- | --- | --- | --- |
| Tree 1 | S1 | 112°55'27" E | 28°10'35" N | 68.66m | 12m | 0.18m | YES |
| Tree 2 | S2 | 112°56'26" E | 28°11'34" N | 53.32m | 18m | 0.67m | YES |
| Tree 3 | S3 | 112°55'42" E | 28°10'52" N | 223.46m | 32m | 1.17m | YES |

**Table S2.** The sampling date and environmental temperature.

| **ID** | **Sampling date** | **Temperature** |
| --- | --- | --- |
| **T1** | April 1st, 2019 | 12.6±0.6℃ |
| **T2** | May 1st, 2019 | 21.3±1.2℃ |
| **T3** | June 1st, 2019 | 25.3±2.3℃ |
| **T4** | July 1st, 2019 | 27.0±0.0℃ |
| **T5** | August 1st, 2019 | 32.2±0.4℃ |

**Table S3.** The dry weight and moisture content of a leaf from T1 to T5 stage.

| **ID** | **T1** | | **T2** | | **T3** | | **T4** | | **T5** | |
| --- | --- | --- | --- | --- | --- | --- | --- | --- | --- | --- |
|  | Fresh weight (g) | Moisture content(%) | Fresh weight (g) | Moisture content (%) | Fresh weight (g) | Moisture content (%) | Fresh weight (g) | Moisture content (%) | Fresh weight (g) | Moisture content (%) |
| S1 | 0.37±0.03 | 83.01±0.42 | 0.55±0.04 | 76.96±0.84 | 0.72±0.06 | 82.87±0.38 | 0.82±0.05 | 78.13±0.54 | 0.87±0.02 | 72.13±0.13 |
| S2 | 0.34±0.02 | 83.12±0.93 | 0.54±0.03 | 77.96±1.05 | 0.75±0.04 | 75.94±1.14 | 0.86±0.05 | 73.89±0.17 | 0.88±0.03 | 68.62±0.46 |
| S3 | 0.46±0.03 | 81.51±1.28 | 0.67±0.07 | 76.67±1.45 | 0.93±0.07 | 79.69±0.26 | 1.03±0.06 | 76.25±0.62 | 1.07±0.02 | 73.02±0.08 |

**Table S4.** ANOSIM results between groups

| ANOSIM | T1 | T2 | T3 | T4 | T5 |
| --- | --- | --- | --- | --- | --- |
| T1 | 0 | 0.001 | 0.001 | 0.002 | 0.001 |
| T2 | 0.8573 | 0 | 0.001 | 0.001 | 0.002 |
| T3 | 0.7596 | 0.3967 | 0 | 0.008 | 0.005 |
| T4 | 0.8954 | 0.7496 | 0.2962 | 0 | 0.013 |
| T5 | 0.6944 | 0.9012 | 0.289 | 0.2414 | 0 |

Note: The values of upper triangular matrices are the significance value (p-value). The values of lower triangular matrices for ANOSIM are R value.

**Table S5.** The relative abundance of endophytic bacterial community in *Ginkgo* leaves at phylum level.

| Phylum | T1 | T2 | T3 | T4 | T5 |
| --- | --- | --- | --- | --- | --- |
| Actinobacteria | 7.86c | 22.75b | 27.26b | 41.09a | 29.65ab |
| Proteobacteria | 26.90a | 16.85bc | 24.40a | 19.83ab | 27.29a |
| unclassified | 19.18a | 16.96ab | 19.34a | 13.87b | 16.17ab |
| Firmicutes | 1.60d | 30.49a | 14.39b | 11.06bc | 8.84c |
| Chloroflexi | 26.20a | 4.33b | 4.12b | 4.37b | 5.00b |
| Cyanobacteria | 14.84a | 6.37b | 5.90bc | 4.25c | 7.99b |
| Bacteroidetes | 2.27ab | 1.59b | 3.39a | 3.99a | 3.49a |
| Acidobacteria | 0.57a | 0.30ab | 0.32ab | 0.32ab | 0.48a |
| Deinococcus-Thermus | 0.18bc | 0.05c | 0.30b | 0.61a | 0.67a |
| Verrucomicrobia | 0.14a | 0.06a | 0.07a | 0.09a | 0.09a |
| CandidatusSaccharibacteria | 0.05a | 0.04a | 0.14a | 0.08a | 0.08a |
| Fusobacteria | 0.03a | 0.01a | 0.04a | 0.20a | 0.06a |
| Gemmatimonadetes | 0.00b | 0.12a | 0.08a | 0.07a | 0.04a |
| Euryarchaeota | 0.01a | 0.01a | 0.21a | 0.00a | 0.00a |
| Spirochaetes | 0.06a | 0.02a | 0.00a | 0.07 | 0.02a |
| Nitrospirae | 0.04a | 0.02a | 0.01a | 0.02a | 0.06a |
| Planctomycetes | 0.03a | 0.01a | 0.00a | 0.05a | 0.00a |
| Armatimonadetes | 0.02a | 0.00a | 0.00a | 0.01a | 0.03a |
| Elusimicrobia | 0.02a | 0.00a | 0.01a | 0.01a | 0.01a |
| Chlamydiae | 0.00a | 0.01a | 0.01a | 0.00a | 0.01a |
| Others | 0.03 | 0.01 | 0.02 | 0.01 | 0.02 |

**Table S6.** Spearman Correlation test showing the relative abundance of endophytic bacterial genera association with three flavonol glycosides.

| Genus | Quercetin | | Kaempferol | | Isorhamnetin | |
| --- | --- | --- | --- | --- | --- | --- |
|  | r | p | r | p | r | p |
| ***Acinetobacter*** | 0.251 | 0.096 | 0.262 | 0.083 | 0.274 | 0.069 |
| ***Actinomadura*** | **0.414** | **0.005** | **0.497** | **0.001** | **0.480** | **0.001** |
| ***Aerococcus*** | **0.407** | **0.006** | **0.503** | **0.001** | **0.527** | **0.000** |
| ***Bacillus*** | 0.012 | 0.937 | 0.022 | 0.884 | -0.048 | 0.754 |
| ***Blastococcus*** | **0.448** | **0.002** | **0.524** | **0.000** | **0.540** | **0.000** |
| ***Chryseobacterium*** | **0.304** | **0.043** | **0.358** | **0.016** | **0.356** | **0.017** |
| ***Corynebacterium*** | 0.019 | 0.901 | 0.109 | 0.475 | 0.093 | 0.542 |
| ***Cupriavidus*** | 0.293 | 0.051 | **0.393** | **0.008** | **0.394** | **0.008** |
| ***Deinococcus*** | **0.411** | **0.005** | **0.502** | **0.001** | **0.508** | **0.000** |
| ***Delftia*** | **0.455** | **0.002** | **0.543** | **0.000** | **0.552** | **0.000** |
| ***Duganella*** | 0.122 | 0.425 | 0.118 | 0.440 | 0.181 | 0.232 |
| ***Elizabethkingia*** | **0.539** | **0.000** | **0.612** | **0.000** | **0.620** | **0.000** |
| ***Enterobacter*** | 0.213 | 0.160 | 0.196 | 0.195 | 0.278 | 0.065 |
| ***Erwinia*** | **0.350** | **0.019** | **0.306** | **0.041** | **0.353** | **0.018** |
| ***Erythrobacter*** | **0.484** | **0.001** | **0.576** | **0.000** | **0.578** | **0.000** |
| ***Geodermatophilus*** | **0.370** | **0.013** | **0.388** | **0.009** | **0.409** | **0.006** |
| ***Limnobacter*** | **0.484** | **0.001** | **0.576** | **0.000** | **0.580** | **0.000** |
| ***Lysinibacillus*** | 0.162 | 0.286 | **0.361** | **0.015** | **0.331** | **0.027** |
| ***Massilia*** | -0.131 | 0.388 | -0.119 | 0.437 | -0.075 | 0.623 |
| ***Methylobacterium*** | **0.360** | **0.016** | **0.392** | **0.008** | **0.447** | **0.002** |
| ***Methyloversatilis*** | **0.476** | **0.001** | **0.558** | **0.000** | **0.565** | **0.000** |
| ***Novibacillus*** | **0.343** | **0.022** | **0.407** | **0.006** | **0.384** | **0.010** |
| ***Oceanobacillus*** | 0.047 | 0.758 | 0.122 | 0.422 | 0.076 | 0.617 |
| ***Planococcus*** | **0.357** | **0.017** | **0.464** | **0.001** | **0.478** | **0.001** |
| ***Prevotella*** | **0.396** | **0.007** | **0.425** | **0.003** | **0.432** | **0.003** |
| ***Propionibacterium*** | **-0.399** | **0.007** | **-0.394** | **0.008** | **-0.394** | **0.008** |
| ***Pseudomonas*** | 0.007 | 0.960 | -0.074 | 0.625 | -0.014 | 0.925 |
| ***Pseudonocardia*** | **0.441** | **0.003** | **0.510** | **0.000** | **0.523** | **0.000** |
| ***Saccharomonospora*** | **-0.360** | **0.016** | **-0.426** | **0.004** | **-0.465** | **0.001** |
| ***Sinibacillus*** | 0.198 | 0.192 | 0.221 | 0.145 | 0.157 | 0.302 |
| ***Sphingomonas*** | -0.293 | 0.051 | -0.172 | 0.257 | -0.152 | 0.318 |
| ***Staphylococcus*** | **-0.334** | **0.023** | -0.258 | 0.087 | -0.250 | 0.097 |
| ***Stappia*** | **0.500** | **0.001** | **0.591** | **0.000** | **0.592** | **0.000** |
| ***Stenotrophomonas*** | **0.486** | **0.001** | **0.553** | **0.000** | **0.593** | **0.000** |
| ***Streptophyta*** | 0.242 | 0.109 | 0.254 | 0.092 | 0.270 | 0.073 |
| ***Thermobifida*** | -0.135 | 0.377 | -0.171 | 0.261 | -0.233 | 0.123 |
| ***Unclassified*** | **0.356** | **0.017** | **0.441** | **0.003** | **0.436** | **0.003** |
| ***Virgibacillus*** | **0.497** | **0.001** | **0.565** | **0.000** | **0.531** | **0.000** |

*Significant differences (*P*<0.05) are indicated in bold.

**Table S7.** Spearman Correlation test showing the relative abundance of endophytic bacterial genera association with physical and chemical parameters.

| Genus | MDA | chlorophyll A | chlorophyll B | SOD | Water | Temperature |
| --- | --- | --- | --- | --- | --- | --- |
| *Acinetobacter* | **-0.361*** | 0.063 | -0.007 | 0.201 | 0.276 | -0.277 |
| *Actinomadura* | 0.098 | -0.124 | -0.137 | **-0.552***** | -0.015 | -0.123 |
| *Aerococcus* | **-0.331*** | 0.120 | 0.008 | -0.151 | -0.087 | 0.238 |
| *Bacillus* | **0.674***** | 0.061 | 0.131 | -0.082 | -0.031 | 0.033 |
| *Blastococcus* | -0.236 | -0.032 | -0.116 | **-0.324*** | -0.041 | 0.115 |
| *Chryseobacterium* | **-0.465**** | -0.022 | -0.098 | -0.201 | **-0.35*** | **0.408**** |
| *Corynebacterium* | -0.195 | 0.135 | 0.106 | 0.209 | **-0.347*** | **0.386**** |
| *Cupriavidus* | **-0.388**** | -0.047 | -0.123 | -0.216 | **-0.381*** | 0.29 |
| *Deinococcus* | **-0.386**** | 0.059 | -0.011 | -0.043 | -0.16 | 0.286 |
| *Delftia* | -0.286 | -0.049 | -0.121 | **-0.492**** | -0.004 | 0.106 |
| *Duganella* | -0.215 | -0.076 | -0.129 | **-0.566***** | 0.011 | 0.026 |
| *Elizabethkingia* | -0.274 | -0.117 | -0.181 | **-0.336**** | -0.125 | **0.431**** |
| *Enterobacter* | -0.147 | 0.002 | -0.050 | **-0.499***** | 0.124 | 0.124 |
| *Erwinia* | 0.018 | 0.032 | 0.016 | -0.220 | -0.044 | 0.176 |
| *Erythrobacter* | -0.270 | -0.060 | -0.134 | **-0.478**** | -0.113 | 0.132 |
| *Geodermatophilus* | **-0.346*** | 0.119 | 0.045 | -0.214 | -0.018 | 0.174 |
| *Limnobacter* | -0.277 | -0.063 | -0.135 | **-0.458**** | **0.392**** | **-0.51***** |
| *Lysinibacillus* | **-0.411**** | -0.190 | **-0.299*** | **-0.383**** | **-0.409**** | **0.349*** |
| *Massilia* | **-0.649***** | 0.036 | -0.028 | -0.009 | **-0.376*** | **0.408**** |
| *Methylobacterium* | -0.281 | 0.197 | 0.119 | 0.177 | -0.143 | **0.419**** |
| *Methyloversatilis* | -0.274 | -0.061 | -0.131 | **-0.470**** | **0.4**** | **-0.596***** |
| *Novibacillus* | 0.277 | -0.140 | -0.127 | **-0.566***** | -0.025 | -0.116 |
| *Oceanobacillus* | 0.188 | 0.068 | 0.093 | **-0.410**** | -0.354 | 0.206 |
| *Planococcus* | -0.214 | -0.022 | -0.090 | **-0.433**** | -0.273 | 0.241 |
| *Prevotella* | -0.293 | 0.110 | 0.084 | -0.049 | -0.015 | 0.068 |
| *Propionibacterium* | -0.244 | 0.134 | 0.132 | **0.720***** | **-0.426**** | **0.503***** |
| *Pseudomonas* | -0.057 | 0.093 | 0.098 | 0.007 | 0.116 | 0.189 |
| *Pseudonocardia* | -0.179 | 0.013 | -0.071 | **-0.439**** | 0.028 | 0.074 |
| *Saccharomonospora* | **0.662***** | **0.403**** | **0.508***** | 0.231 | -0.175 | 0.197 |
| *Sinibacillus* | **0.638***** | -0.024 | 0.036 | **-0.359*** | -0.026 | -0.061 |
| *Sphingomonas* | **-0.753***** | 0.265 | 0.155 | **0.398**** | **-0.485**** | **0.42**** |
| *Staphylococcus* | -0.171 | 0.201 | 0.179 | **0.569***** | -0.163 | **0.38**** |
| *Stappia* | -0.246 | -0.073 | -0.145 | **-0.483**** | -0.002 | -0.094 |
| *Stenotrophomonas* | -0.252 | -0.224 | **-0.305*** | **-0.474**** | 0.204 | 0.05 |
| *Streptophyta* | **-0.395**** | **-0.318*** | **-0.354*** | **-0.360*** | **0.371*** | **-0.545***** |
| *Thermobifida* | **0.656**** | 0.012 | 0.112 | -0.028 | -0.062 | 0.138 |
| *Virgibacillus* | 0.030 | -0.021 | -0.052 | **-0.495**** | 0.073 | 0.062 |

*Indicated significant differences (*P*<0.05); ** Indicated significant differences (*P*<0.01); ***Indicated significant differences (*P*<0.001).

**Table S8.** RDA results

| Env | r^2^ | p |  |
| --- | --- | --- | --- |
| SOD | 0.4211 | 0.001 | *** |
| Chlorophyll_A | 0.0445 | 0.384 |  |
| MDA | 0.3079 | 0.002 | ** |
| Quercetin | 0.2361 | 0.004 | ** |
| Kaempferol | 0.4111 | 0.001 | *** |
| Water | 0.3316 | 0.001 | *** |
| Temprature | 0.5328 | 0.001 | *** |

**Table S9.** Mantel test

| Envs | r | p |
| --- | --- | --- |
| SOD | 0.3661 | 0.001 |
| Chlorophyll_A | 0.1268 | 0.033 |
| MDA | 0.1704 | 0.012 |
| Quercetin | 0.3642 | 0.001 |
| Kaempferol | 0.3386 | 0.001 |
| Water | 0.2616 | 0.001 |
| Temprature | 0.5455 | 0.001 |

**Table S10.** Species classification and draft genomes details.

| ID | Similarity (%) | Phylum | Genus | Strain Name | Genbank No. |
| --- | --- | --- | --- | --- | --- |
| S1 | 96.87 | Actinobacteria | *Microbacterium* | *Microbacteriumtestaceum* N106 | MK629778 |
| S2 | 94.75 | Deinococcus-Thermus | *Deinococcus* | *Deinococcussoli* N5 | KU992913 |
| S3 | 96.82 | Firmicutes | *Paenibacillus* | *Paenibacillustyrfis* PK2-1.1 | MN428207 |
| S4 | 95.63 | Firmicutes | *Bacillus* | *Bacillusmegaterium* TSS4 | MF620065 |
| S5 | 96.91 | Firmicutes | *Bacillus* | *Bacilluscereus* ML208 | KC692193 |
| S6 | 96.29 | Firmicutes | *Bacillus* | *Bacillussubtilis* KSU_B17 | MN208475 |
| S7 | 96.04 | Firmicutes | *Bacillus* | *Bacillusvelezensis* N308 | MK629801 |
| S8 | 97.08 | Firmicutes | *Staphylococcus* | *Staphylococcusxylosus* N202 | MK629787 |
| S9 | 97.41 | Proteobacteria | *Achromobacter* | *Achromobacterxylosoxidans* SMF3 | AJ880396 |
| S10 | 96.43 | Proteobacteria | *Burkholderia* | *Burkholderiacepaciastrain* KSB-32 | MK280757 |
| S11 | 98.33 | Proteobacteria | *Massilia* | *Massiliahaematophila* L-2 | KU305723 |

# Supplementary Figures

# Supplementary Figure 1. Rarefaction curves.


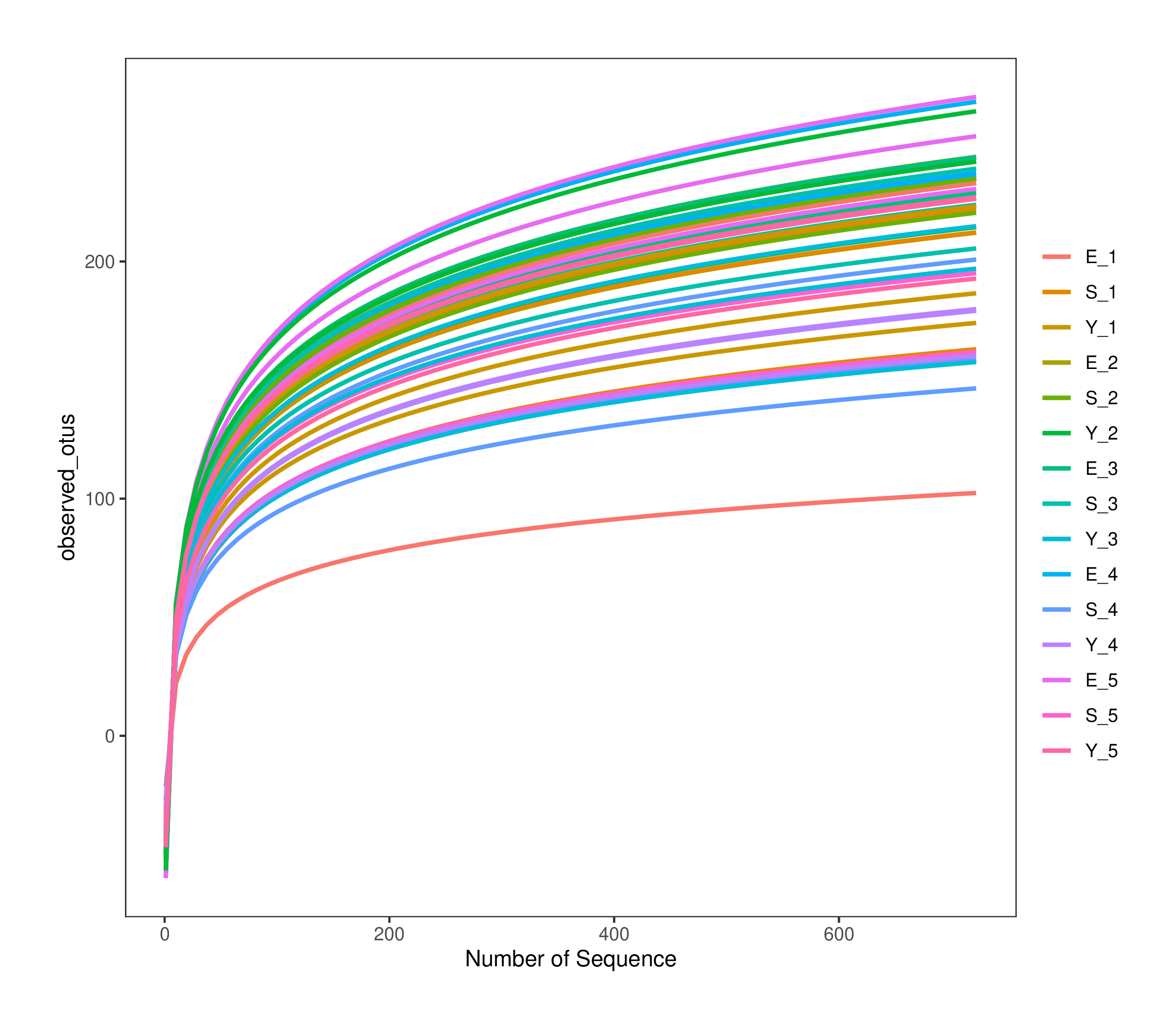


**Supplementary Figure 2.**Venn diagram.


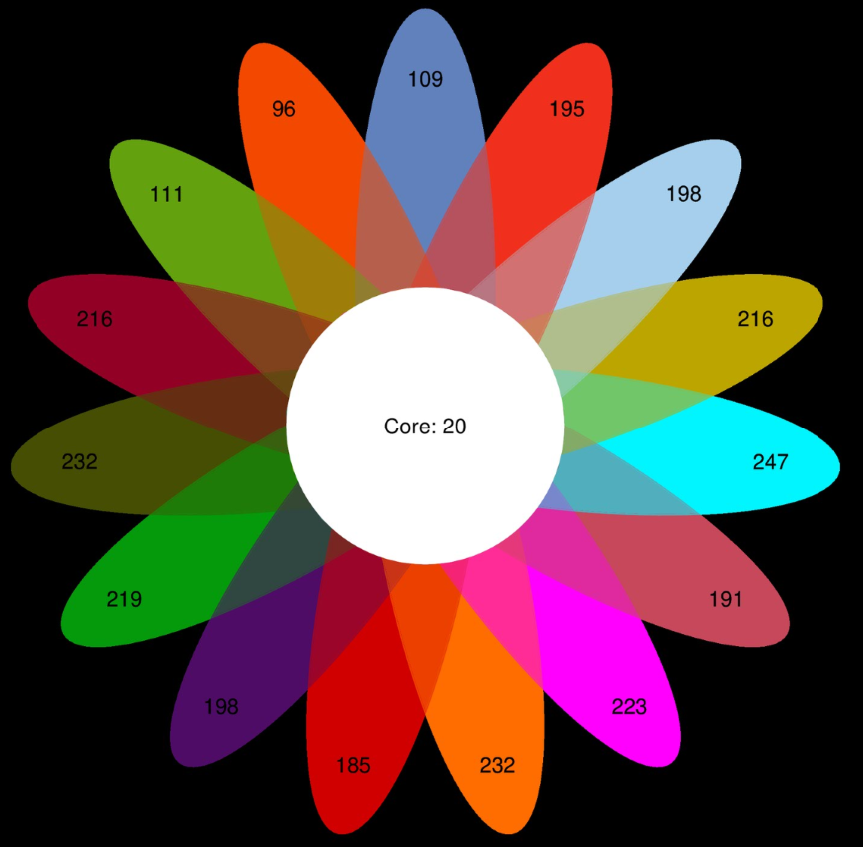


**Supplementary Figure 3** Endophytic bacterial relative abundance at the phylum level**.**


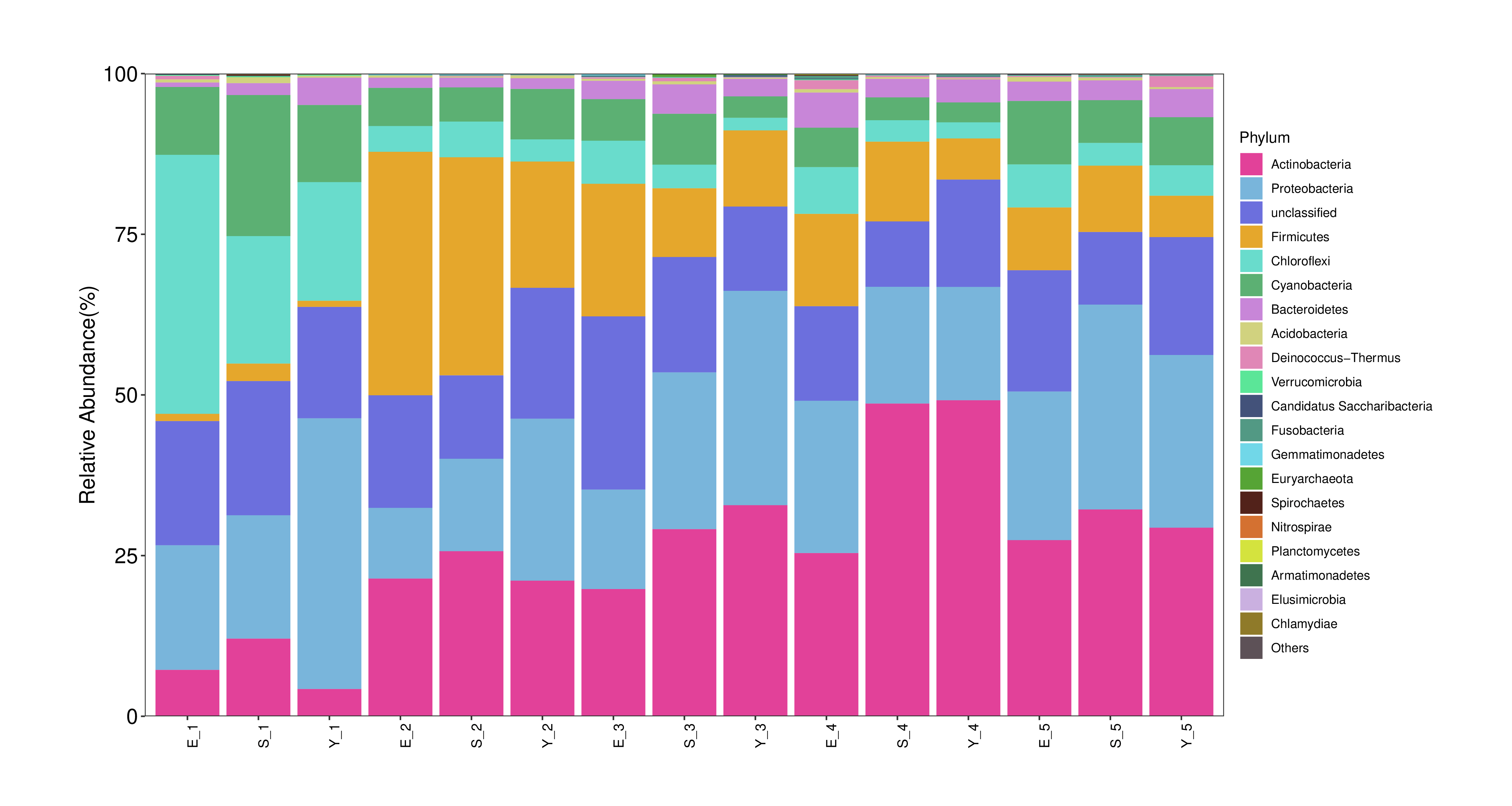


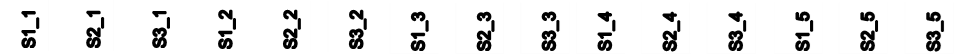


**Supplementary Figure 4** Endophytic bacterial relative abundance at the genus level**.**


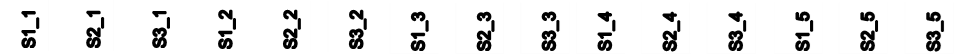

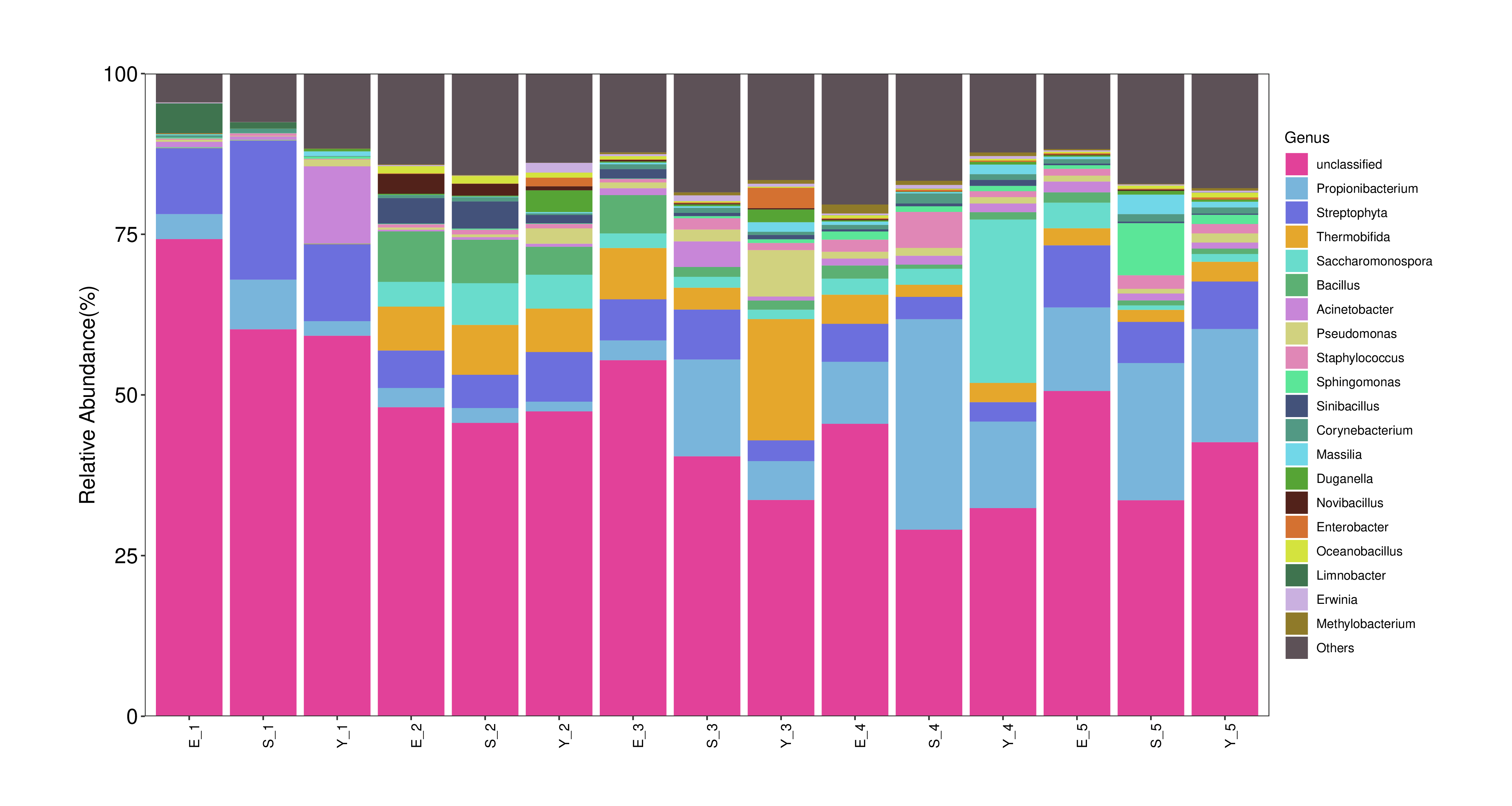

Supplement: Supplementary file 1 [file Data_Sheet_1.docx]
